# Annual Research Review: A multilevel bioecological analysis of factors influencing the mental health and psychosocial well‐being of refugee children

**DOI:** 10.1111/jcpp.13355

**Published:** 2020-12-05

**Authors:** Stella Arakelyan, Alastair Ager

**Affiliations:** ^1^ Institute for Global Health and Development Queen Margaret University Edinburgh UK

**Keywords:** Refugee, children, mental health, stressors, protective factors, psychosocial support

## Abstract

**Background:**

This paper revisits the themes of an influential 1993 review regarding the factors shaping the mental health and psychosocial well‐being of refugees to take stock of developments in the evidence base and conceptualisation of issues for refugee children over the last 25 years.

**Methods:**

The study deployed a systematic search strategy. This initially identified 784 papers, which was reduced to 65 studies following application of inclusion and exclusion criteria. We used a later iteration of Bronfenbrenner’s bioecological model of human development – the PPCT model – to consolidate evidence.

**Results:**

We identify a range of risk and protective factors operating at individual, familial, community and institutional and policy levels that influence outcomes for refugee children. The dynamics shaping the interaction of these influences are linked to the life course principles of socio‐historical time and developmental age, proximal processes and child agency.

**Conclusions:**

Actions at individual, familial, community, school, institutional and policy levels all have potential traction on mental health and psychosocial well‐being of refugee children. However, evidence suggests that greatest impact will be secured by multilevel interventions addressing synergies between ecological systems, approaches engaging proximal processes (including parenting programmes) and interventions facilitating the agency of the developing refugee child.

## Introduction

Of the estimated global refugee population of approaching 26 million refugees, over a half are under the age of eighteen (UNHCR, 2018). The ‘global refugee crisis’ of the last decade has been marked by large‐scale displacement of populations from conflict‐affected and low‐income settings and varieties of accommodation and resistance of these movements by other nations. Factors such as displacement experience, familial separation and acculturative stress all have clear implications for children’s well‐being and development outcomes in such circumstances. However, interpretation of the recent wave of research into these processes is usefully viewed from the perspective of lessons learned – and conceptual frames established – in the course of multiple prior periods of population displacement with major implications for children.

In 1993, the second author completed a review of mental health in refugee populations with a developmental focus on behalf of Harvard Centre for the Study of Culture and Medicine (Ager, 1993). This was part of the preparation for the production of *World Mental Health: Problems and Priorities in Low‐Income Settings* (Desjarlais, Eisenberg, Good, & Kleinman, 1995), one of the foundational works in establishing the field of global mental health. That review serves as a useful guide to the refugee situations of concern twenty‐five years ago, the bodies of research literature related to these concerns and – of particular interest here – the conceptual framing of that literature.

In the mid‐1990s, the refugee displacements of central concern were related to the sequelae of the Vietnam War, the Cambodian genocide, proxy wars in southern Africa and the unfolding violence of the Balkans with the break‐up of the former Yugoslavia. Accordingly, the research literature reviewed in Ager (1993) was heavily shaped by the acculturative processes of South‐East Asian migrants to the United States (US), along with the rise of the refugee camp as a temporary – but increasingly protracted – setting for children’s development.

There were two major themes structuring the Ager (1993) literature review: one regarding the analysis of risks and protective or ameliorative factors associated with the nested layers of resource – from familial process through religious or political affiliation – shaping individual adaptation and development; the other, the sequential nature of the refugee experience through phases of preflight, flight, reception and (re)settlement. Although not explicitly cited, these principles very much reflect Bronfenbrenner’s conceptualisation of developmental influence (Bronfenbrenner, 1979, 1995), most particularly in the form of the later iteration of his PPCT – person‐process‐context‐time model (Bronfenbrenner and Evans, 2000; Bronfenbrenner, 2005).

In the PPCT model (Bronfenbrenner and Evans, 2000; Bronfenbrenner, 2005), Bronfenbrenner elaborates his established analysis of multi‐layered contextual systems (i.e. micro‐, meso‐, exo‐ and macro‐ systems) with respect to the person, proximal processes and time. Bronfenbrenner sees persons as embodying a multiplicity of biological and genetic mechanisms which collectively impact on behaviour and development (Bronfenbrenner, 2005). Progressively more complex reciprocal enduring interactions – proximal processes – operate between the developing child and ‘significant others’, objects and symbols in their microsystem to serve as ‘the engines of development’ (Bronfenbrenner & Evans, 2000, p. 118). Within the PPCT model, time reflects both ‘historical time’ and ‘developmental age’. A person’s life course is embedded in and shaped by both the circumstances and events happening in ‘the historical period in which one lives’ and ‘biological and social transitions based on culturally defined age, role expectations, and opportunities’ (Bronfenbrenner, 1995, p. 641).

**Figure 1:**
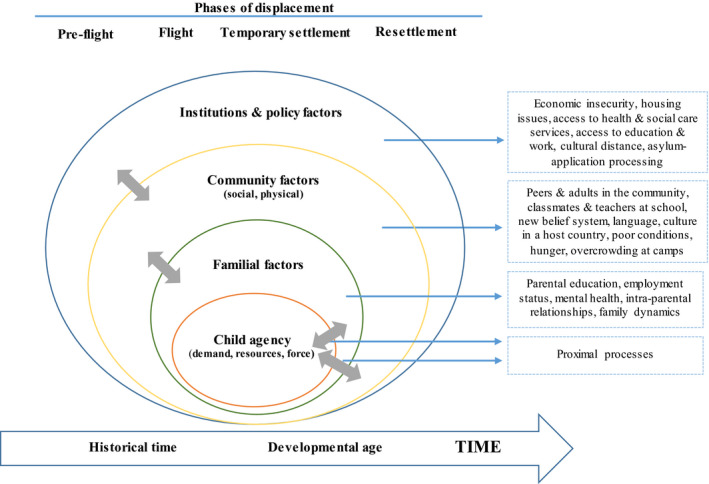
Simplified version of Bronfenbrenner’s person‐process‐context‐time model applied to the child refugee experience

Reflecting on the suitability of this framing for studies of forced migration, Hayes and Endale (2018) note how the model’s focus on ‘examining multiple levels of context and change over time…can be especially useful in examining the complex issues surrounding the development of identity in newcomer children and adolescents’ (p. 285). It is thus the framing device for the current view (Figure 1), providing a coherent basis to assess our advance over the last 25 years in understanding of the factors and processes shaping the mental health and psychosocial well‐being of refugee children.

## Methods

A review protocol (available from the first author) was developed *a priori* specifying search strategy, search terms and keywords, inclusion and exclusion criteria, data extraction, data synthesis and analysis.

### Search strategy and screening

A systematic search was undertaken in five databases – MEDLINE (EBSCO), CINAHL (EBSCO), PsycINFO (EBSCO) and SCOPUS (Pro‐Quest) for peer‐reviewed evidence published in English since January 2010 up to November 2019. Restriction by publication date was applied to capture the evidence reflective of the effects of recent political and war‐driven displacement in Middle East, Asia, Sub‐Saharan Africa and Latin America. Search terms and keywords were developed in consultation with a subject‐specialist librarian. The following combination of terms were used: child OR minor* OR young OR adolescent OR youth OR teenage AND asylum seeker OR refuge* OR displaced person OR internally displaced person AND psychiatr* OR psycholog* OR psychosocial well‐being OR psychosocial wellbeing OR mental OR mental health OR mental issue OR distress OR stress OR emotional stress OR emotion AND risk factor* OR stressor* OR trauma OR vulnerability factor* AND protective factor* OR adaptation* OR modifying factor* OR resilience OR develop* OR social support* OR coping* OR psychosocial support* OR recovery OR wellbeing OR well‐being OR adjustment OR behavio?r OR outcome.

### Selection criteria

Studies were included if (a) the primary aim was to explore risks (stressors) and/or protective factors/processes contributing to mental health and psychosocial well‐being of refugee children and (b) study participants were refugee children (age range 0–18 years or mean age < 18 years) or caregivers sharing information about their refugee children. A risk factor was defined as a psychosocial adversity or event that would be considered a stressor to most people and that may hinder normal functioning (Masten, 1994). Protective factors were defined as exogenous variables whose presence is associated with desirable outcomes in populations deemed at risk for mental health (Werner, 1989). Protective processes were operationalized as dynamic processes operating in the family, peer group, school and community which tend to buffer the likelihood of negative outcomes (Benard, 1995). The WHO’s definition of mental health was adopted: ‘a state of well‐being in which every individual realizes his or her own potential, can cope with the normal stresses of life, can work productively and fruitfully and is able to make a contribution to her or his community’ (WHO, 2004). Only original, peer‐reviewed published articles were included. Studies were excluded if (a) more than 50% of participating children and young people had mean age > 18 and where no segregated data by age were presented; (b) they focused principally on describing the feasibility or effectiveness of mental health‐promoting interventions; and (c) they provided a literature review and no primary data. No restriction to a country was applied.

### Data extraction

Data extraction was performed using a standardized data extraction form. Details were extracted on (a) the study design, (b) population demographics, (c) setting and length of stay, (d) methods of data collection, (e) factors intrinsic to the child and (f) factors related to ecological systems of potential influence in Bronfenbrenner’s bioecological model (Bronfenbrenner, 1979, 1995, 2005). Reporting categories for the latter were developed iteratively through the process of data extraction, resulting in distinction of familial, community (social and physical), and institutions and policy systems.

## Results

Initial database search identified 784 articles. On removal of duplicates, 519 titles and abstracts were screened for relevance resulting in 86 full‐text articles retrieved for further eligibility assessment. Sixty‐six articles corresponding to 65 individual studies met the inclusion criteria. Summaries of the included studies are presented in Tables 1 and 2.

**Table 1:** Summary of included quantitative studies

**First author (year)**	**Study type**	**Population**			**Location**	**Length of stay (µ ± *SD*)**	**Individual factors**	**Familial factors**	**Community factors**		**Institutions and policy factors**
		**Characteristics**	**Age (µ ± *SD*)**	**Sample size**					**Social factors**	**Physical factors**	
Aitcheson (2017)	CSS	Palestinian adolescents in refugee camps	17–19 (µ = 17.4)	335	Gaza, Palestine		Age, optimism, ethnic identity, self‐regulation, coping skills	Family coherence			
Afifi (2019)	CSS	Palestinian adolescents residing in two refugee camps	11–21	185	Beirut, Lebanon		Personal uncertainty	Interparental conflict, communal coping			
Akiyama (2013)	CSS	Burmese adolescent students living in boarding houses	12–18 (µ = 16)	428	Tak province, Thailand		Gender, No of traumatic events	Parental depression, parenting style, poverty, family functioning	Discrimination from peers and adults		Time in Thailand
Beiser (2015)	CSS	Immigrant children and refugees	11–13	2,074	Six Canadian cities		Age, gender, instrumental competence, social competence, cultural distance, acculturation strategies, resettlement stress				Time in Canada
Beiser (2016)	CSS	Refugee youth	11–13	326‐immigrants, 152‐refugees	Six major urban areas in Canada	≤10 y	Gender, ethnicity, feel welcomed at school, predisplacement human and social capital	Depression, mother report of premigration trauma, poverty			
Bryant (2018)	CS	Refugees admitted to 11 sites in Australia	Caregiver 25–45, children 5–18	Caregivers 394, children 639	11 sites across Australia			Trauma history, postmigration difficulties, greater PTSD, harsh parenting			
Buchegger‐Traxler (2012)	CSS	Austrian adolescents and migrant adolescents	14–19	1100	Vienna & Linz, Austria	~10 y	Age, gender, exposure to violence, religion, neighbourhood attachment, social distance	Intergenerational conflict, parental monitoring, family connectedness	Peer support, school connectedness		
Çeri (2018)	CSS	Syrian refugee minors	7–17	85	Hatay, Turkey	(29.8 m ± 11.2 m)	Age, gender, No of traumatic events, having a parent with maltreatment/ torture history, not feeling satisfied with resettlement, having a familiar person left behind, witnessing insult/others killed, seeing corpses	Less educated fathers			
Clukay (2019)	CSS	Syrian refugees	12–18	399	Irbid, Jarash, Mafraq, Zarqa, Jordan		Gender, MAOA‐L genetic variant, resilience				
Correa‐Velez (2010)	CSS	Refugee youth	12–18	97	Melbourne, Australia		Age, gender, country of birth, subjective social status	Living with parents	Peer attachment, peer bulling, discrimination		Time
Eruyar (2018)	CSS	Syrian refugee children	8–18 (11.6 ± 1.86)	263	Istanbul, Turkey	0 m–6 y (2.09 y ± 1.01 y)	Age, gender, exposure to trauma	Parental psychopathology, parenting‐related stress			
Eruyar (2020)	CSS	Syrian refugee minors	8–17 (11.6 ± 1.81)	322	Istanbul, Turkey		Age, gender	Insecure attachment, negative parenting style, secure parental and maternal attachment, parenting styles			
Hanes (2019)	CSS	Asylum seekers	(6 ± 4.72)	110	Western Australia			Family separation			Lack of service access, prolonged detention, interrupted education
Hirani (2018)	CSS	Unaccompanied refugee minors	15–18 (16.95 ± 0.82)	41	Austria		Feelings of anger, ongoing anxiety				
Khamis (2019)	CSS	Syrian refugee children and adolescents	7–18 (11.30 ± 2.65)	1,000 (500 from Lebanon, 500 from Jordan)	Lebanon, Jordan	1–72 m (48.6 ± 15.6)	Age, gender, No of war atrocities, coping styles	Family expressiveness	School context	Type of host country	Time
Khan (2019)	CSS	Rohingya children	0–16 (7.4 ± 3.6)	662	Myanmar, Bangladesh		Parentless	Family member killed/died, type of housing, parental education			
Jani (2016)	CSS	Unaccompanied immigrant youth	9–18 (µ = 15.5)	138	United States	1.03–44.2 m (µ = 10.43 m)		No of experienced abuses in their home countries			
Jensen (2014)	CS	Unaccompanied refugee children	arrived <15 y. (16.5 ± 1.6)	75	Norway	T1 6 m + T2 1.9 y (0.9–2.8 y)	Age, gender, length of stay, length of education				
Karam (2019)	CSS	War‐exposed Syrian refugee children and adolescents	7–17 (11.9 ± 1.6)	549	Lebanon	2/3 <5 y, 1/3>5 y	Age, gender, childhood adversities, war exposure, sensitivity				
Longobardi (2017)	CSS	Unaccompanied minors from Egypt, Albania, Senegal, Bangladesh, Gambia, Morocco and Mali	16–17	19	Italy		Multiple traumas, religion	Separation from parents	Peer issues		
Mace (2014)	RS	Resettled refugee children	4–18 (9.58 ± 3.43)	332	Western Australia	(6.63 ± 5.09 m)	Symptoms of distress, PTSD, depression, primary nocturnal enuresis, poor appetite, symptoms of nightmares, separation anxiety, aggressive behaviour	Family separation, single parent (mother) as guardian			Incorrectly documented, multiple migration experiences, mandatory detention, residing in community detention
Lincoln (2016)	CSS	Young refugees from Somalia	11–20 (15.39 ± 2.2)	135	3 New England cities, United States	1–14 y (5.40 ± 3.32)	Age, No of traumas, higher severity of acculturation hassles, acculturation style				Time in the US
Mels (2010)	CSS	War‐affected Eastern Congolese adolescents	13–21	819	Ituri district, the Democratic Republic of Congo		Age, gender, traumatic exposure, daily stressors, IDP status	Death of mother and death of father, death of father had an effect on externalizing symptoms			
Montgomery (2010)	LS	Young refugees from the Middle East	11–23 (µ = 15.3)	131	Denmark	8–9 y	No of traumatic exposures, No of types of stressful events after arrival	Length of father’s education in the home country, ‘speaks frequently with mother about problems’		Young refugee attending school or work	Time
Nasıroğlu (2018)	CSS	Children and adolescents living in a refugee camp in Turkey	6–17 (11.05 ± 3.11)	136	Beşiri district, Batman, Turkey		Age, witnessing war and violence, suffered from violence in Iraq, witnessing a gunfight, people getting injured during migration	Parents with psychiatric illness history			Time in the camp
Oppedal (2012)	CSS	Unaccompanied minor asylum seekers	13–27 (18.9 ± 2.64)	566	Norway	(µ = 3.7 y)	Age, gender, in‐group competence, in‐group hassles, outgroup competence, outgroup hassles				Time
Oppedal (2015)	CSS	Unaccompanied minors resettled in Norway	(18.6 ± 2.51)	895	Norway	(3.45 y ± 2.28 y)	Culture competence	Social support from the family	Peer networks		
Oppedal (2018)	CSS	Syrian refugee children living in camp in Turkey	µ = 12.5	285	Turkey	Just over 5 m in the refugee camp	Gender, accumulation of traumatic events	Changes in family	Social support		
Panter‐Brick (2015)	LS	Afghan youth	11–16 (13.23 ± 1.55)	331	Kabul, Afghanistan and the Afghan refugee camps in Peshawar, Pakistan	One‐year follow‐up	Gender, lifetime trauma exposures	Family level domestic violence, mother literacy, poverty, father literacy, illiterate mother, a caregiver with improving mental health	Neighbourhood interactions	Live in Peshawar refugee camps	
Patel (2017)	CSS	War‐exposed newcomer adolescents	9–12 grades	184	United States	0–8 y (µ = 3.5 y)	Exposure to war, daily/acculturative life stressors				
Sangalang (2017)	LS	Southeast Asian refugee mothers and their children	Mothers (42 ± 7.6),children (12 ± 1.1)	327	United States	(13.6 ± 5.31)	Ethnicity, nativity	Maternal traumatic distress, family functioning			
Sapmaz (2017)	CSS	Children and their families (Syria, Iraq, Afghanistan, Iran)	5–18 (9.96 ± 3.98)	89	Turkey	(22.1 m ± 13 m)	Age, witnessing a dead or injured individual, positive history of a psychiatric disorder in the child	Father’s educational level, father’s employment status, positive history in the family for a psychiatric disorder			Homeless, hunger
Sim (2018)	CSS	Syrian refugee mothers	Mothers (31.8 ± 8.18), children 2–12 (7.44 ± 3.23)	291	Lebanon		Exposure to daily stressors	Mothers’ general psychological distress, post‐traumatic stress, negative parenting			
Sleijpen (2016)	LS	Adolescent refugees and asylum seekers	12–17 (14.5 ± 1.8)	111	The Netherlands	(µ >3 y)	Perceived level of social support, dispositional optimism, refugee status				
Van Ee (2012)	CSS	Asylum‐seeker, refugee mothers and their children	Mothers 19–44 (29.5 ± 6.2), children: 16–46 m (26.6 m ± 8.3 m)	49	The Netherlands	Asylum seekers (2.7 y ± 1.1 y); refugee (8.1 y ± 4.8 y)		Maternal post‐traumatic stress symptoms, maternal emotional availability within mother–child interaction			
Van Ee (2013)	CSS	Refugee and asylum‐seeking families	Fathers µ = 35.6, mothers µ = 29.6, children µ = 27.14 m	80	The Netherlands	(8.64 y ± 5.22 y)		Father–child relationship			
Veronese (2017)	CSS	Palestinian children living in refugee camps	6–11 (8.8 ± 1.42)	1,276	The Gaza Strip – Bureij, Gaza Beach Camp, Jabalia, Rafah		Age, gender, negative emotions, subjective well‐being				
Veronese (2012)	CSS	Palestinian children	9–11 (10.8 ± 1.9)	216	Tulkarem Region, the West Bank		Pessimism, gender , age, optimism, life satisfaction, perceived happiness				
Vervliet (2014)	LS	Unaccompanied refugee minors	14–17 (15.9 ± 0.85)	103	Belgium	T1‐arrival, T2–6 m, T3–18 m	No of daily stressors, No of traumatic experiences, gender				Time
Völkl‐Kernstock (2014)	CSS	Unaccompanied refugee minors from Africa	15–18, (16.95 ± 0.82)	41	Austria		Gender, frequencies of trauma exposure, feelings of danger and revenge, coping strategies				
Zwi (2018)	LS	Newly arrived refugee children	4–15	43	Australia	T1‐year 2 (µ = 11 m; 5–24 m), T2 year 3 (µ = 31 m, 21–40 m)	No of stressful events, fewer stressful life events in the previous year, number of protective factors, proximity to one’s own ethnic community, having relatives in Australia prior to arrival	Originated from Eastern Mediterranean region, absent fathers, originated from Africa, father present on arrival, settlement factors	Support from the general community		Time

CSS, cross sectional study; LS, longitudinal study; RS, retrospective study

[Correction made on 11 December 2020, after first online publication: The alignment errors and data inconsistencies in Table 1 have been corrected in this version.]

**Table 2:** Summary of included mixed methods and qualitative studies

**First author (y)**	**Study type**	**Population**			**Location**	**Length of stay** (µ ± *SD*)	**Individual factors**	**Familial factors**	**Community factors**		**Institutions and policy factors**
		**Characteristics**	**Age** (µ ± *SD*)	**Sample size**					**Social factors**	**Physical factors**	
Bermudez (2018)	QS	Adolescents in Kiziba Camp	13–19	70	Rwanda		Engaging in risky coping behaviours, transactional sex	Crowded familial living arrangements, intergenerational conflict, parent–child relationship		Overcrowding, far travel to collect firewood, food insecurity, unemployment, security issues in the camp	Economic insecurity, resource constraints, lack of housing, general security issues
Bettmann (2018)	MM	Refugee youths resettled in the US	15.9	37	Western US city	(4.4 y ± 3.0)	Secure attachment	Age, nonproductive coping, reference to other as a coping strategy – ‘pray’, seeking to belong, gender			
Cameron (2018)	MM	Refugee children attending mainstream and specialist language schools	12–18 (14.8 ± 1.6)	80	Metropolitan region, Melbourne	1 m–15 y (4.7 y ± 5.6)	Coping strategies				
Chase (2013)	QS	Unaccompanied young people seeking asylum in the UK	11–23	54	London, the UK		Destabilising impact of trauma on self, lack of status, loss of identity, insecurity, re‐emergence of insecurity, need for order, routine and security, learning language, education /learning				
Dalgaard (2017)	MM	Families from Iraq, Iran, Lebanon, Palestine, Syria and Afghanistan	Children 4–9	30	Denmark			Parenting style, family flexibility, family roles, stressor pile‐up, problem‐solving skills/conflict level, family functioning, family cohesion, parental coping strategies			
Dalgaard (2019)	MM	Families referred to rehabilitation centres due to trauma‐related family violence	children <18	21	Denmark			Problematic or dysfunctional parent–child interaction			
Ellis (2010)	MM	Somali adolescent refugees	11–20 (µ = 15.4)	135	Three New England cities	1–14 y (µ = 5.4 y)	Social identity, acculturation		Peer discrimination		
Eriksson (2019)	QS	Unaccompanied refugee minors (boys) from Afghanistan, Syria and Somalia	arrived 14–16, current 17–20	10	Sweden	2–5 y	A lack of acceptance, identity, having a sense of belonging, learning language, internal coping strategies – maintaining calmness, reflecting	Reunification with family, parents provide practical and financial support	Social safety, social interaction, support, emotional safety, active listening, verbal strategies, adaptive mental strategies		
Groark (2011)	QS	Unaccompanied asylum‐seeking children and adolescents	16–18	6	The UK	at least 6 m	Loss, impact of evaluation by others, experience of distress, negotiating a new way of life, process of adjustment				
Hayes (2018)	QS	Refugee adolescents	arrived 7–16 (µ = 11), interviewed 18–40 (µ = 30)	18	United States		Impact of conflict or living situation – important for sense of identity, newcomer identity		Impact on adaptation processes, conflicting cultural values between microsystems, language and academic issues		
Kienzler (2019)	QS	Kosovar adolescents deportees	15–18	14	European countries		Social isolation, lack of help‐ and health‐seeking behaviour, coping strategies	Economic problems, health problems of other family members, family support	Discrimination, harassment, local friends, friends in the host country	Precarious living conditions	Deportation and arrival back to Kosovo, housing problems, lack of professional support and doctors
Lauritzen (2012)	QS	Refugee children living at governmental asylum processing centres	not specified	Staff 34	Northern Norway			Parents' helplessness, parental mental health problems which affect parenting style	Isolation, segregation, poverty, feeling included and being part of a community, schooling, activities	Housing conditions, safety of staff and residents	Life and time at the asylum centre, the lack of competence of centre workers, shortage of staff, international and national policies and events, basic rights
Jarlby (2018)	QS	Unaccompanied refugee minors	17–18	6	Rural Denmark	2–3 y (µ = 1.5 y)	Belonging to social network, coping strategies, practicing religion, social, physical activities, schooling and employment opportunities, learning language, contact with family, social support				
McFarlane (2011)	QS	Resettled refugee children and young people, parents, counsellor‐advocates and managers	13–22	26 (12 children, 4 parents, 10 staff)	Australia		Subjective emotional states and observable behaviour: post‐traumatic stress, depression, grief, anxiety, distress and worry, somatic signs and symptoms, loneliness, loss of hope, withdrawal, isolation, distrust, feeling sad, survival guilt	Family functioning lack of parental guidance, family nurturance, disrupted sense of belonging, lack of adult support, ‘parentification’ of a young person caring for siblings, problems in a new family	Unfamiliarity with the new environment, difficulties with communication, rejection due to bullying or discrimination, making connections		
McGregor (2016)	QS	Refugee adolescents	12–21 (17 ± 2.6)	43	Hobart and Melbourne, Australia	6 m–11 y (µ = 4.4 y)	Sense of belonging, religious values, meaning systems, moral values, beliefs, identity/role system	Family as a source of stress, family separation, family attachment, family relationships	Lack of peer relations, hard to establish friendships, peer attachment relationships	Extended social circle attachment relationships	Safety, the justice system
Meyer (2013)	QS	Refugee children from Burma	Adults 18–50, children 9–17	25 adults and 23 children/youth	Ban Mai Nai Soi camp, Thailand		Risky behaviours, mental health issues (worries, sad, lonely), early marriage, early pregnancy	Adults behaviours (drinking, fighting each other), financial problems, neglecting and bullying children	Education problems		
Meyer (2019)	QS	Adolescent refugees from South Sudan	13–17	325 (183 adolescents and 142 caregivers)	Kiryandongo and Adjumani, Uganda					Influx of new refugees brought food insecurity, overcrowding, violence, injury, lack of access to education	
Sim (2018)	QS	Syrian refugees (parents and children)	Parents 18–60, (µ = 31.3), children (µ = 9.2)	39 Syrian parents and 15 children	Lebanon		Child emotional and behavioural problems	Diminished parenting quality –parental supervision, lower quality parent–child interaction, harsh parenting, parental control, parental mental health, positive parenting, social support from spouse	Bullying and abuse from Lebanese teachers, social isolation, social support	Economic hardship, lack of safety pathway	Economic resources
Veronese (2015)	QS	Palestinian children from Nur Shams and Tulkarm refugee camps	7–15 (10.8 ± 2.06)	74	The West Bank – Nur Shams, Tulkarm		Play, agency and self‐determination, positive affect, satisfaction of primary needs	Family providing protection, a safe environment, economic well‐being	Relationships with peers and adults		Religion, sociality and access to education
Veronese (2017)	QS	Palestinian children living in refugee camps	6–11 (8.8 ± 1.4)	200	The Gaza Strip – Bureij, Gaza Beach Camp, Jabalia, Rafah		Negative emotions, gender, child agency, activism, self‐satisfaction, coping strategies, religious expression, play, personal resources, satisfaction with school, friends and living environment	Family providing protection, a safe environment	Social positive relations, peers		Environmental insecurity, a lack of resources, access to school
Veronese (2018)	QS	Palestinian children living in refugee camps	6–15 boys (10.3 ± 0.6), girls (10.9 ± 0.3)	122	The West Bank and the Gaza Strip		Negative emotions, gender political wellbeing, personal growth, positive emotions	Social positive relations within family			Barriers to accessing school, constraints to freedom of movement, human security, access to school
Stark (2015)	QS	Congolese and Somali adolescents, caregivers, service providers	13–17	175	Kampala, Uganda		Perceived parental and community support	Indirect discrimination through impact on family, powerless parents/no resources to justice, discrimination in job market/hiring, a withdrawal from help‐seeking behaviour or community participation	Peer discrimination in school/neighbourhood settings		Lack of access to post‐rape medical, mental health, other supportive services
Thommessen (2015)	QS	Unaccompanied asylum‐seeking minor males from Afghanistan	18–19 (arrived as 15–16)	6	Sweden		Living in limbo, striving to fit in and move forward	Lack of contact with family members, lack of knowledge about their wellbeing	Social support and positive encouragement, mentoring, friendships with other youth who had experienced similar difficulties		

QS, qualitative study; MM, mixed methods study.

[Correction made on 11 December 2020, after first online publication: The alignment errors and data inconsistencies in Table 2 have been corrected in this version.]

The majority of studies were conducted in high‐income contexts: Scandinavia (10), Australia (10), the United States (7), mainland Europe (9), Canada (2) and the United Kingdom (2). Nineteen studies were focused in the Middle East: including Palestine (7), Turkey (6), Jordan (2) and Lebanon (5). Four studies were based in Africa and two in Asia. Forty‐one studies (63.1%) employed quantitative, 19 (29.2%) qualitative and 5 (7.7%) mixed‐method study designs, varying substantially in terms of sample size (range 6–2,074), participants’ ages (children aged 0–23, caregiver aged 18–60), and methods of data collection (e.g. surveys, in‐depth interviews, focus groups, observations, drawing). Thirteen (20%) studies reported on unaccompanied refugee children; eight (61.5%) of which presented quantitative data. Two qualitative articles used the same study participants, but different data collection methods (Veronese & Cavazzoni, 2019; Veronese, Cavazzoni, & Antenucci, 2018). Evidence supplied by these articles was partially overlapping and is therefore reported as a single study.

Included studies describe factors and processes contributing to refugee children’s mental health and psychosocial outcomes in relation to multiple adversities faced at the individual, familial, community, and institutions and policy levels. Argumentative synthesis of these descriptions is presented below.

### Individual factors

#### Age

Higher rates of psychotrauma and emotional dysregulation are reported in children who are adolescents (Buchegger‐Traxler & Sirsch, 2012; Correa‐Velez, Gifford, & Barnett, 2010; Khamis, 2019; Nasıroğlu, Çeri, Erkorkmaz, & Semerci, 2018; Panter‐Brick, Grimon, Kalin, & Eggerman, 2015; Sapmaz et al., 2017). This may be associated with various factors, for example prolonged exposure to adversities or compromised family or peer relationships (Çeri & Nasıroğlu, 2018). Buchegger‐Traxler and Sirsch (2012) found lower levels of perceived neighbourhood attachment, family and school connectedness in older children – factors positively contributing to children’s mental health and psychosocial well‐being. In three studies, however, younger age was a risk factor (Aitcheson, Abu‐Bader, Howell, Khalil, & Elbedour, 2017; Beiser, Puente‐Duran, & Hou, 2015; Lincoln, Lazarevic, White, & Ellis, 2016) with older children presenting as more psychologically resilient (Aitcheson et al., 2017). In one study, younger Syrian children are reported as being exposed to less traumatic events, but presenting with more mental health problems, although these were mainly predicted by parental psychopathology rather than age *per se* (Eruyar, Maltby, & Vostanis, 2018).

#### Gender

Psychomorbidity including emotional problems, depression, anxiety and somatic symptoms are more prevalent in girls (Akiyama et al., 2013; Buchegger‐Traxler & Sirsch, 2012; Çeri & Nasiroğlu, 2018; Nasıroğlu et al., 2018; Oppedal, Özer, & Şirin, 2018; Panter‐Brick, Grimon, Kalin, & Eggerman, 2015; Vervliet, Lammertyn, Broekaert, & Derluyn, 2014). This is linked to such factors as ‘non‐productive’ coping strategies (Cameron, Frydenberg, & Jackson, 2018), higher levels of dissatisfaction with living conditions, feelings of hopelessness for the future (Nasıroğlu et al., 2018) and lower neighbourhood attachment (Buchegger‐Traxler & Sirsch, 2012). This is despite boys being exposed to more traumatic events, at least in public settings, than girls (Eruyar et al., 2018). One study found a monoamine oxidase A genetic variant (MAOA – a gene proposed to influence the impact of childhood trauma on adult violence and antisocial behaviour) to play a role in influencing perceived psychosocial stress amongst Syrian refugee boys, independent of their trauma exposure, but not in girls (Clukay et al., 2019). Five studies, four of which included large samples (i.e. sample size ranging between 549 and 1,000), found no association between gender and mental health (Jensen, Skårdalsmo, & Fjermestad, 2014; Karam et al., 2019; Khamis, 2019; Khan et al., 2019; Mels, Derluyn, Broekaert, & Rosseel, 2010), albeit Mels et al. (2010) found that girls are more likely to report internalising symptoms when experiencing high levels of daily stressors in post‐resettlement.

#### Ethnicity & ethnic or national identity

While strength of ethnic and national identity varies amongst groups of refugees (d’Abreu, Castro‐Olivo, & Ura, 2019), this is frequently found to be a protective factor for refugee children. A stronger sense of ethnic and national identity is associated with less depression, anxiety and more resilience for Palestinian children residing in camps in Gaza (Aitcheson et al., 2017). Similarly, a strong sense of ethnic identity contributed to positive psychosocial well‐being in resettled young refugees in Australia (Correa‐Velez et al., 2010; Zwi et al., 2018). Somali adolescent girls presented with better mental health outcomes if having a stronger ethnic identity and felt more protected from discrimination when surrounded by peers sharing common ethnicity (Ellis et al., 2010). However, ethnic and national identity may interact with acculturation strategy. It was Somali adolescent boys who assimilated within mainstream culture in host cities in New England who presented with better mental health (Ellis et al., 2010). Being identified with a minority ethnic group can exacerbate anti‐refugee discrimination by peers in the host community, with deleterious mental health consequences (Beiser & Hou, 2016).

#### Acculturation style

The minority of refugee children who are given an opportunity to resettle in a third country have to adjust to new belief system, culture, language and try to meet country‐specific academic expectations (Ellis et al., 2010). For many children, these expectations present major additional ‘acculturation stresses’ (Eriksson & Rundgren, 2019; Oppedal & Idsoe, 2012; Patel et al., 2017). These stresses are generally conceptualised in terms of a cultural conflict between heritage and host cultures (Berry, 1992) which requires adaptation. In general, the higher the perceived level of acculturation stress, the more depressive symptoms children exhibit (Oppedal & Idsoe, 2012; Patel et al., 2017).

Acculturation style mediates the relationship between acculturation stress and mental health outcomes (Beiser & Hou, 2016; Beiser et al., 2015; Oppedal & Idsoe, 2012; Thommessen, Corcoran, & Todd, 2015). Children adopting an integrative acculturation style, which assumes interest in and adherence to heritage culture alongside an active incorporation of values and attitudes from the host culture, are widely found to report better mental health outcomes (Beiser et al., 2015; Eriksson & Rundgren, 2019; Oppedal & Idsoe, 2012; Thommessen et al., 2015). Adopting an acculturation style based on separation or marginalization contributes to persistent challenges and poor mental health (Beiser & Hou, 2016; Beiser et al., 2015).

An integrative acculturation style requires instrumental and social competencies (Beiser et al., 2015; Oppedal & Idsoe, 2012). These competencies refer to the perceptions of self as able to perform school‐related tasks, and make and retain social relationships (Beiser et al., 2015). Perceptions of instrumental (e.g. fluency in host country language) and social competencies (e.g. forming friendships, getting along with others) are associated with increased self‐esteem, self‐efficacy, feelings of mastery and lower levels of perceived discrimination (Oppedal & Idsoe, 2012, 2015). These competences mitigate the adverse effect of cultural distance (i.e. the distinctiveness of culture of origin and that of resettlement) and social distance (i.e. low acceptance of relations with other ethnic groups) on mental health and promote child psychosocial well‐being (Beiser et al., 2015; Oppedal & Idsoe, 2012, 2015).

Host country language literacy in particular plays an important role in children’s social and cultural competencies. Children obtaining fluency in the host country language report better functioning within new social networks and school systems (Eriksson & Rundgren, 2019). This can however put a strain on parent–child relationships; since children typically learn host country language and customs quicker than parents, parents may use their children as translators and ‘cultural interpreters’ (Lauritzen & Sivertsen, 2012).

#### Coping style

Psychosymptomology and emotional dysregulation vary according to children’s coping styles (Aitcheson et al., 2017; Çeri & Nasiroğlu, 2018; Eriksson & Rundgren, 2019; Groark, Sclare, & Raval, 2011; Jarlby, Goosen, Derluyn, Vitus, & Jervelund, 2018; Khamis, 2019; Veronese, Castiglioni, Tombolani, & Said, 2012; Veronese, Pepe, Jaradah, Muranak, & Hamdouna, 2017a; Veronese, Pepe, Jaradah, Murannak, & Hamdouna, 2017). Palestinian children residing in urban districts, rural areas and a refugee camp in the West Bank displayed positive adjustment despite facing socio‐economic disadvantage and military violence (Aitcheson et al., 2017; Veronese et al., 2012). Ability to remain optimistic, happy and resistant in the face of multi‐adversity contributed to a high level of life satisfaction and positive mental health functioning (Jarlby et al., 2018; Veronese et al., 2012; Veronese, Pepe, Jaradah, Al Muranak, & Hamdouna, 2017; Veronese, Pepe, Jaradah, Murannak, et al., 2017). Aitcheson et al., (2017) found that nearly half of Palestinian refugee youth living in camps in Gaza presented as resilient, showing minimal signs of depression, display strong competence in self‐regulation and having optimistic world view. Many studies document the role of increased utilization of effective problem‐focused coping (e.g. problem solving, seeking social support, opposition to adversity) in promoting better mental health outcomes (Afifi et al., 2019; Eriksson & Rundgren, 2019; Groark et al., 2011; Khamis, 2019; Jarlby et al., 2018; Veronese, Pepe, Jaradah, Al Muranak, et al., 2017; Veronese, Pepe, Jaradah, Murannak, et al., 2017), as well as the corollary of emotion‐focused coping (e.g. avoidance, social withdrawal, self‐criticism, dissatisfaction) leading to poorer outcomes (Çeri & Nasiroğlu, 2018; Eriksson & Rundgren, 2019; Khamis, 2019).

#### Religion

Religion is widely noted as a central to refugee children’s lives, positively shaping and providing a frame to consolidate their experience (Buchegger‐Traxler & Sirsch, 2012; Jarlby et al., 2018; Longobardi, Veronesi, & Prino, 2017; McGregor, Melvin, & Newman, 2016; Veronese, Pepe, Jaradah, Murannak, et al., 2017). Unaccompanied refugee minors from a wide range of settings seeking refuge in host countries referred to religion as a central source of their strength and protection, calling on their faith for help when experiencing daily challenges (Jarlby et al., 2018; Longobardi et al., 2017). In Australia, refugee youth were found to draw upon moral and religious beliefs to provide a direction, sense of meaning and purpose in life (McGregor et al., 2016).

#### Risky behaviour

Engagement in antisocial behaviour, alcohol and substance use contributes to feelings of depression amongst refugee children (Bermudez, Parks, Meyer, Muhorakeye, & Stark, 2018; Buchegger‐Traxler & Sirsch, 2012; Stark, Plosky, Horn, & Canavera, 2015). This is more pronounced in males, facilitated by higher neighbourhood attachment and peer support from the host community (Buchegger‐Traxler & Sirsch, 2012). Alcohol abuse, transactional sex, night outs with ‘sugar daddies’ has been reported as prevalent in females living in camps (Stark et al., 2015) with clear risks for unintended pregnancies or early marriages (Bermudez et al., 2018). Engagement in risky behaviours is widely seen as a consequence of economic hardship (Bermudez et al., 2018). However, children’s narratives indicate that compromised parent–child relationships and parental inability to provide tangible resources may exacerbate such risks (Stark et al., 2015).

### Familial factors

#### Parental education & employment

The impacts of parental education or employment status on child mental health are addressed by relatively limited number of studies with inconsistent results (Khan et al., 2019; Panter‐Brick et al., 2015; Sapmaz et al., 2017). Positive association was established between maternal (but not paternal) literacy levels and Afghan children’s distress levels (Panter‐Brick et al., 2015). Maternal literacy is however rare in the Afghan context (Panter‐Brick et al., 2015). It assumes certain family dynamics and many psychosocial pressures on children (e.g. schooling, employment) from literate mothers following the loss of maternal salaried employment and persistent poverty (Panter‐Brick et al., 2015). In a study of refugees in Turkey children’s psychopathology was linked with paternal education and paternal unemployment status (Sapmaz et al., 2017). No association was found between parental education and mental health of Rohingya children in Myanmar (Khan et al., 2019).

#### Parental mental health

Studies consistently report that parental post‐traumatic symptoms, anxiety and depression negatively affect children’s mental health and psychosocial adjustment (Nasıroğlu et al., 2018). This happens via two main mechanisms (a) indirectly through an ‘inefficient’ parenting style (Dalgård & Montgomery, 2017; Eruyar, Maltby, & Vostanis, 2018, 2020; Lauritzen & Sivertsen, 2012; Panter‐Brick et al., 2015; Van Ee, Sleijpen, Kleber, & Jongmans, 2013); and (b) directly by making children anxious, worried and saddened (Dalgård & Montgomery, 2017; Lauritzen & Sivertsen, 2012). Findings across several studies indicate that traumatised parents present as short‐tempered, irritable, impatient or violent towards their children. These parenting practices, commonly described as ‘punitive’ or ‘harsh’, are consistently linked to poor child mental health outcomes (Bryant et al., 2018; Dalgård & Montgomery, 2017; Eruyar, Maltby, & Vostanis, 2018, 2020; Panter‐Brick et al., 2015; Van Ee et al., 2013). Many parents report guilt, regret and hopeless in their lack of competence to maintain an emotional bond (Dalgård & Montgomery, 2017; Van Ee et al., 2013; Lauritzen & Sivertsen, 2012; Sim, Bowes, & Gardner, 2018) or to nurture and provide children with age‐appropriate structured routines (Dalgaard, Thøgersen, Væver, & Montgomery, 2019), compromising their perceptions of agency (Lauritzen & Sivertsen, 2012).

Parents are commonly aware of negative effects of traumatic experience on their children’s psychosocial well‐being and seek to adopt strategies to mitigate these effects (Van Ee et al., 2013; Sim, Fazel, Bowes, & Gardner, 2018). Parents speak of putting on ‘brave front’ for their children and neglecting their own needs so that children do not feel deprived (Van Ee et al., 2013; Sim, Fazel, et al., 2018). Many report decreased emotional availability and sensitivity, withdrawal from family life and separation at times of emotional turmoil (Dalgård & Montgomery, 2017; Dalgård et al., 2019). These active coping strategies are not always effective (Dalgård & Montgomery, 2017). However, in general, improved parental mental health predicts decline in children’s distress levels (Panter‐Brick et al., 2015). To assist parents in overcoming mental health challenges, some children reciprocate the parental care‐giving role by offering comfort, food and drink or physical proximity (Dalgård & Montgomery, 2017). This parent‐child role reversal, or so‐called ‘childhood parentification’, is perceived as an added emotional burden on children (Dalgård & Montgomery, 2017).

#### Parent–child interaction

Family is a key source of social support for children. Evidence consistently supports the role of family ties as an asset and positive parent–child relationships as a source of strength, protection and security for children (Bermudez et al., 2018; Bettmann & Olson‐Morrison, 2018; Eruyar, Maltby, & Vostanis, 2020; Lauritzen & Sivertsen, 2012; McGregor et al., 2016; Nasıroğlu et al., 2018; Oppedal & Idsoe, 2015; Veronese & Castiglioni, 2015; Veronese et al., 2018; Veronese, Pepe, Jaradah, Murannak, et al., 2017; Zwi et al., 2018) Unaccompanied refugee children who have contact with their families living abroad perceive high levels of support and present with lower levels of depression, despite lack of physical contact and face‐to‐face communication with families (Eriksson & Rundgren, 2019; Jani, Underwood, & Ranweiler, 2016; Oppedal & Idsoe, 2015).

Scholarly consensus is that supportive and sensitive child–parent interactions are key to children’s emotional and behavioural development. Yet, the family and parent–child interactions can be perceived as a source of added stress (Buchegger‐Traxler & Sirsch, 2012; Dalgård & Montgomery, 2017; McGregor et al., 2016; Sim, Fazel, et al., 2018). For example, while fathers may report the value of positive parent–child interaction, there may find it difficult to control their anger and irritability prompted by the uninterrupted flow of daily stressors (Van Ee et al., 2013; Sim, Fazel, et al., 2018). The range of reported stressed impacting parent–child interaction includes family traumatic histories; parental mental health problems and physical illness; financial hardship; perceived discrimination and abuse from host neighbours, employers, healthcare providers, and authorities; worries about the extended family in the country of origin; and uncertainty with residency permits or citizenship in a host country (Dalgård & Montgomery, 2017; Van Ee et al., 2013; Sim, Fazel, et al., 2018).

Strained parent–child relationships, commonly described in literature as ‘intergenerational conflict’, have a negative effect on children’s emotional well‐being and psychosocial adjustment (Dalgård & Montgomery, 2017; Van Ee et al., 2013). Communication problems are a widely reported source of distress in these circumstances (Afifi et al., 2019; Bermudez et al., 2018). Refugee children feel unable to openly discuss their feelings or report incidences of violence in the community or at school (Afifi et al., 2019; Bermudez et al., 2018). Bermudez et al., (2018) found that children’s disclosure of sexual violence from adults in a camp in Rwanda rarely resulted in parental use of formal reporting and referral processes due to fears of shame, embarrassment and social rejection. Parental awareness of child abuse often led to little or no consequences for the abuser undermining children’s sense of safety (Bermudez et al., 2018). At times, children avoid discussing their struggles and disclosing incidents of abuse due to fearing physical punishment from their parents (Bermudez et al., 2018) or due to concerns over parental mental health problems (Dalgård & Montgomery, 2017) or marital conflict (Afifi et al., 2019).

#### Family cohesion

Family cohesion is understood as the emotional bond family members have with one another (Olson, Russell, & Sprenkle, 1983), involving openness, expressiveness, togetherness and communication. Family cohesion can act as a buffer regarding daily stressors and uncertain coping strategies and social support (Khamis, 2019). A stronger sense of family cohesion (Aitcheson et al., 2017) and open and free expression of negative feelings within a family (Khamis, 2019) have both been associated with reduced psychopathology in refugee children.

Marital cohesion refers to the degree of connectedness and emotional bonding between spouses (Olson et al., 1983). Social support from a spouse promotes positive parenting and reduces the risk of child maltreatment (Sim, Fazel, et al., 2018). Marital cohesion is, however, often at risk in situations of chronic adversity (Afifi et al., 2019). Sharing feelings of uncertainty or disclosing stressors to a family reinforces distress in children in the presence of marital conflict (Afifi et al., 2019). Poor family functioning and marital conflict are linked with child depressive symptoms, antisocial and delinquent behaviour (Afifi et al., 2019), which can persist over time (Sangalang, Jager, & Harachi, 2017).

#### Separation from a family

The presence of psychopathology in refugee children is associated with both current and previous nuclear family separation (Eriksson & Rundgren, 2019; Jani et al., 2016; Longobardi et al., 2017; Mace, Mulheron, Jones, & Cherian, 2014; McGregor et al., 2016; Thommessen et al., 2015) and parentless status (Khan et al., 2019; Zwi et al., 2018). Unaccompanied refugee children frequently speak of the emotional difficulty separation from family brings into their lives (Eriksson & Rundgren, 2019; Jani et al., 2016; McGregor et al., 2016). Connection or unification with the family is viewed as a key source of social support and emotional safety (Eriksson & Rundgren, 2019; Jani et al., 2016; McGregor et al., 2016). Feelings of missing, and being constantly worried about, family members left behind negative impacts on children’s psychosocial well‐being (McGregor et al., 2016; Thommessen et al., 2015). Only one study found no association between the whereabouts of parents, social supports and child mental health (Akiyama et al., 2013).

### Community factors

#### Social factors

For refugee children, mutually affectionate and supportive relationships are described as central for emotional well‐being (Correa‐Velez et al., 2010; Eriksson & Rundgren, 2019; Groark et al., 2011; Jarlby et al., 2018; Lauritzen & Sivertsen, 2012; McGregor et al., 2016; Zwi et al., 2018). Nevertheless, it appears that the mental health effects of relationships and supports are complex, contested and context‐specific (Buchegger‐Traxler & Sirsch, 2012; Chase, 2013; Lauritzen & Sivertsen, 2012).

Refugee children clearly distinguish between the type of relationships they have with a host community (peer, adults) and those sharing a common ethnic and refugee background (Beiser & Hou, 2016; Ellis et al., 2010; Eriksson & Rundgren, 2019; McGregor et al., 2016). Friendships with peers and adults sharing similar ethnic background are much valued and seen as emotionally fulfilling, partly due to common cultural values and traditions shared (Buchegger‐Traxler & Sirsch, 2012; Eriksson and Rundgren, 2019; McGregor et al., 2016; Oppedal & Idsoe, 2015; Thommessen et al., 2015; Zwi et al., 2018). Most importantly, these relationships contribute to children’s sense of belonging and social cohesion (Eriksson & Rundgren, 2019; McGregor et al., 2016; Oppedal & Idsoe, 2015; Thommessen et al., 2015). Friendships with local peers from a host community are seen as critical for ‘fitting in’ with the host community and at school, and improving language proficiency (Chase, 2013; Eriksson & Rundgren, 2019; McGregor et al., 2016; Oppedal & Idsoe, 2015). These host relationships, however, have a potential to negatively affect children’s self‐esteem and facilitate antisocial behaviour and substance use (Buchegger‐Traxler & Sirsch, 2012).

The issues of peer tensions, discrimination, bullying and abuse are regularly raised by refugee children resettled in both temporary settlement and resettlement contexts (Beiser & Hou, 2016; Ellis et al., 2010; Correa‐Velez et al., 2010; Eriksson & Rundgren, 2019; Lauritzen & Sivertsen, 2012; Longobardi et al., 2017, Sim, Fazel, et al., 2018; Stark et al., 2015; Vervliet et al., 2014). The most frequently cited reasons for discrimination include ethnicity, nationality, religion, age, gender, language, ‘newcomer’ or ‘unwelcome guest’ status and clothing (Beiser & Hou, 2016; Ellis et al., 2010; Eriksson & Rundgren, 2019; Lauritzen & Sivertsen, 2012; Sim, Fazel, et al., 2018; Stark et al., 2015; Vervliet et al., 2014). Higher levels of peer discrimination are linked to higher levels of psychomorbidity (Ellis et al., 2010; Lauritzen & Sivertsen, 2012; Sim, Fazel, et al., 2018). Perceptions of discrimination might partly explain why social distancing from peers in the neighbourhood can contribute to better psychosocial and educational outcomes at resettlement (Buchegger‐Traxler & Sirsch, 2012).

#### Physical factors

Poverty, poor hygiene and sanitation, overcrowding, food insecurity and lack of health care and support staff are reported frequently as key stressors linked to children’s poor mental health, especially in those living in camp settings (Bermudez et al., 2018; Khamis, 2019; Lauritzen & Sivertsen, 2012; Meyer, Meyer, Bangirana, Mangen, & Stark, 2019; Nasıroğlu et al., 2018; Panter‐Brick et al., 2015; Sim, Fazel, et al., 2018). The severity of these problems variates by country context (Khamis, 2019; Nasıroğlu et al., 2018; Sim, Fazel, et al., 2018). For instance, a higher prevalence of psychopathology amongst Syrian refugee children was found for those displaced in Lebanon compared to Jordan (Khamis, 2019). This was partially attributed to the more precarious living conditions faced by refugee children in the former setting, including poor sanitation facilities and overcrowded shelter, contributing to a prolongation of adverse experiences after displacement. Further, length of stay in a refugee camp in Beşiri district, Turkey located next to a river polluted by untreated sewage and with dysfunctional facilities predicted poorer mental health amongst Yazidi refugee children (Nasıroğlu et al., 2018).

Narratives of children and their families frequently highlight the safety and security risks for children posed by overcrowded familial living arrangements and lack to access to economic resources (Bermudez et al., 2018; Meyer et al., 2019; Sim, Fazel, et al., 2018). Overcrowding may force girls to seek shelter with non‐family members in unsupervised housing, placing them at risk of physical and sexual violence (Bermudez et al., 2018). Lack of access to economic resources and poverty perpetuates food insecurity, homelessness, involvement in risky behaviour, including sex work, and inability to plan for the future (Bermudez et al., 2018; Sim, Fazel, et al., 2018).

### Institutions and policy factors

#### Prolonged detention and deportation

Children entering a host country for temporary settlement or resettlement, especially those who are unaccompanied or without required documentation, must negotiate the legal system and advocate for their rights. A lengthy asylum process may result in children’s prolonged and multi‐site mandatory detention (Chase, 2013; Hanes, Chee, Mutch, & Cherian, 2019; Lauritzen & Sivertsen, 2012; Mace et al., 2014; Thommessen et al., 2015). Hanes et al. (2019) note that 97% of asylum‐seeking children in Australia report experiencing prolonged detention (median 7 months, range 3–12.5 months) across multiple sites (median 2, range 1–3). Mandatory detention is strongly and consistently associated with poor mental health outcomes and behavioural problems (Hanes et al., 2019; Lauritzen & Sivertsen, 2012; Mace et al., 2014; Thommessen et al., 2015). Reports from children highlight the needs for prompt and fair asylum‐application processing as uncertainties surrounding application outcomes and prospects of deportation are linked to elevated distress levels, worry, and anxiety (Chase, 2013; Thommessen et al., 2015).

Deportation also has major mental health consequences (Chase, 2013; Kienzler, Wenzel, & Shaini, 2019). Children deported back to their countries of origin report peer discrimination, social association, issues with accessing employment, resources and professional help, economic hardship, housing problems and precarious living conditions (Chase, 2013; Kienzler et al., 2019), with immediate family the only source of emotional and practical support in times of their sadness, distress and emotional pain (Kienzler et al., 2019).

#### Access to education and work

Access to education or work is central for refugee children’s mental health and psychosocial well‐being (Buchegger‐Traxler & Sirsch, 2012; Groark et al., 2011; Jarlby et al., 2018; Lauritzen & Sivertsen, 2012; Montgomery, 2010; Meyer et al., 2019; Veronese, Pepe, Jaradah, Murannak, et al., 2017). It allows children to have a break from ‘ordinary’ everyday life, advance their knowledge and better integrate into communities (Groark et al., 2011; Jarlby et al., 2018; Lauritzen & Sivertsen, 2012). Lack of access to education or high rates of interrupted schooling is often reported (Hanes et al., 2019; Lauritzen & Sivertsen, 2012; Meyer et al., 2019; Veronese & Cavazzoni, 2019; Veronese et al., 2018). Access to schooling, while strongly encouraged, also has the potential to undermine children’s sense of safety (Sim, Fazel, et al., 2018; Stark et al., 2015). For example, Syrian refugee children attending Lebanese schools reported experiencing bullying and physical abuse from both Lebanese schoolmates and teachers (Sim, Fazel, et al., 2018).

#### Access to health and support services

Refugee children generally present with complex medical and psychosocial needs (Hanes et al., 2019). They require quick access to health care and support services which is often lacking (Groark et al., 2011; Hanes et al., 2019; Meyer et al., 2019; Sim, Fazel, et al., 2018; Vervliet et al., 2014). Children who have had access to support services highlight the important role support and encouragement from staff play in helping them to overcome anxiety and worries while waiting for an asylum decision (Eriksson & Rundgren, 2019; Groark et al., 2011; Thommessen et al., 2015). Unaccompanied refugee children often keep regular contact with support staff after they leave detention centres (Groark et al., 2011; Eriksson & Rundgren, 2019).

#### Length of asylum and resettlement processes

Time spent in a host country of permanent resettlement is most commonly associated with decreased psychopathology (Khamis, 2019; Montgomery, 2010; Akiyama et al., 2013; Bettmann & Olson‐Morrison, 2018; Correa‐Velez et al., 2010). Montgomery (2010) report that the prevalence of mental health problems decreased from about three‐fourths to one‐fourth at follow‐up in Middle Eastern refugee children resettled in Denmark. However, mental health problems in unaccompanied refugee children resettled in the Netherlands (Jensen et al., 2014) and Belgium (Vervliet et al., 2014) persisted over time, linked to lengthy asylum procedures and resettlement stresses. Resettlement stresses brought by cultural differences (e.g. belief system, culture, language) are frequently reported as the main reasons contributing to instances of deterioration of the mental health of refugee children in Canada over time (Beiser et al., 2015).

## Discussion

As noted earlier, we used a later iteration of Bronfenbrenner’s bioecological model of human development – the PPCT model (Bronfenbrenner and Evans, 2000; Bronfenbrenner, 2005) ‐ to consolidate evidence from the last decade and assess our advance in understanding of the factors shaping mental health and psychosocial outcomes in refugee children since the Ager (1993) review. The findings above indicate significant advance in documenting risk and protective factors associated with specific bioecological strata impacting on the child. However, in addition to characterisation of much of the evidence base on factors predicting psychological adjustment as ‘anecdotal and impressionistic’, the earlier review noted that ‘the complex manner in which such influences may interact is poorly understood’ (p. 23). This is thus potentially another important aspect of strengthened evidence. One of the core propositions of PPCT is the dynamic interaction of individual child factors and multi‐layered contextual factors (see Figure 1). Another is the role of time – in various forms – shaping the child’s interaction with these ecosystemic strata. In this discussion, we therefore develop analysis of the literature identified, and the understanding of the complex processes which shape refugee child mental health and well‐being, by drawing upon these principles. Specifically, we consider the implications of Bronfenbrenner’s understanding of life course principles (socio‐historical time and developmental age), proximal processes and child agency (demand, resource, force characteristics) in determining outcomes (Bronfenbrenner, 1995, 2005).

### Time: historical time and development age

Bronfenbrenner gives a prominent place to life course principles in human development – ‘historical time’ and ‘developmental age’ (Bronfenbrenner, 1995). The original (Ager, 1993) review considered the outworking of historical time in the lives of refugee children with reference to phases of the refugee experience: preflight, flight, temporary settlement and resettlement (for a minority, in a third, host country). This represents a temporal as well as geographical trajectory, although the emergence of widespread protracted displacement in countries of initial refuge (where, as Qasmiyeh (2020) has noted ‘the camp is the incinerator of time’) has rendered the phrasing ‘temporary’ problematic.

Evidence collated from studies highlights the experience of multiple atrocities in preflight phase of displacement. Common reports include experience of armed conflict, political persecution, financial hardship, homelessness, physical violence, torture or sexual abuse, maltreatment, torture or loss of a family member (Akiyama et al., 2013; Chase, 2013; Çeri & Nasiroğlu, 2018; Groark et al., 2011; Jani et al., 2016; Longobardi et al., 2017, Mace et al., 2014; Nasıroğlu et al., 2018; Panter‐Brick et al., 2015; Patel et al., 2017; Sapmaz et al., 2017; Zwi et al., 2018). A dose–response association is often reported whereby children experiencing multiple and prolong exposure before displacement present with more severe PTSD, depression and anxiety (Chase, 2013; Çeri & Nasiroğlu, 2018; Panter‐Brick et al., 2015; Sapmaz et al., 2017; Vervliet et al., 2014). Differences are also identified in relation to type of trauma exposure (Nasıroğlu et al., 2018; Sapmaz et al., 2017). Children who suffer physical violence, witness gunfights and people being killed are more likely to present with more severe PTSD (Nasıroğlu et al., 2018; Sapmaz et al., 2017).

Many children continue to be exposed to violence, hunger, physical or sexual abuse, forced labour, homelessness during flight (Groark et al., 2011; Longobardi et al., 2017; Nasıroğlu et al., 2018; Sapmaz et al., 2017; Vervliet et al., 2014). Those experiences are especially prominent in unaccompanied refugee children being frequently exploited by traffickers. In the study by Longobardi et al., (2017), all unaccompanied refugee minors (19) reported experiencing physical abuse (e.g. being insulted, hit with an object, punished with infliction of pain, tortured, forced to engage in dangerous activity) and more than half sexual abuse. These children reported up to a 6‐month long migration journey (Longobardi et al., 2017).

Although recent literature has continued to document exposure to deprivations and stressors associated with the preflight and flight phases (and the psychological sequelae of these), the most marked development in the evidence base over the last 25 years is with respect to the stressors of settlement (either temporary – or protracted – within camps or urban settings) and resettlement (most commonly through formal resettlement or asylum processes). There is strong evidence regarding the impacts of these (Ellis et al., 2010; Beiser & Hou, 2016; Çeri & Nasiroğlu, 2018; Groark et al., 2011; Montgomery, 2010; Patel et al., 2017; Thommessen et al., 2015; Vervliet et al., 2014). Host countries often fall short in providing adequate psychosocial, health care, educational and financial support to refugee children (Çeri & Nasiroğlu, 2018; Hirani, Cherian, Mutch, & Payne, 2018; Mace et al., 2014; Montgomery, 2010; Patel et al., 2017; Thommessen et al., 2015; Vervliet et al., 2014). Dissatisfaction and emotional tensions arising from uncertainly, unmet needs and expectations are linked to a range of persistent psychosocial problems (Çeri & Nasiroğlu, 2018; Groark et al., 2011; Hirani et al., 2018; Jensen et al., 2014; Montgomery, 2010; Sim, Fazel, et al., 2018; Zwi et al., 2018). Again, these experiences are more pronounced for unaccompanied minors, who are variably accommodated in the care system in different countries (Hirani et al., 2018; Jensen et al., 2014). For instance, a two‐year follow‐up assessment of unaccompanied refugee children’s mental health resettled in Norway showed that symptoms of psychopathology were overall unchanged over time, with 11% scoring high on suicidal ideation (Jensen et al., 2014).

This axis of historical time interacts with developmental age. Younger children are generally at greater risk for developing psychopathology in response to war‐related adversities (Aitcheson et al., 2017; Beiser et al., 2015; Lincoln et al., 2016). This is because infants and young children rely heavily on parents (or other caregivers) for food, warmth, shelter and protection (Smith, Cowie, & Blades, 2015). They also depend on early positive parent–child interaction for development of social skills and cultural meaning. Provisions of these resources are heavily compromised amongst refugee families, especially in those at temporary settlements. Evidence however suggests higher rates of PTSD and depression amongst older children (Buchegger‐Traxler & Sirsch, 2012; Correa‐Velez et al., 2010; Khamis, 2019; Nasıroğlu et al., 2018; Panter‐Brick et al., 2015; Sapmaz et al., 2017). Again, this trend is especially apparent in those who are unaccompanied (Hirani et al., 2018; Jensen et al., 2014; Longobardi et al., 2017).

The experience of older children often includes prolonged and cumulative adversities, including ongoing community violence, sexual abuse, parental neglect and repeated domestic violence, abuse of power by those in authority (Çeri and Nasiroğlu, 2018; Longobardi et al., 2017; Nasıroğlu et al., 2018; Sapmaz et al., 2017). This type of repeated, at times life‐threatening, experiences typically undermines basic trust in others and their social attendance resulting in breakdown of social interactions (proximal processes) which tend to protect against the development of psychosocial problems (Çeri & Nasıroğlu, 2018).

### Proximal processes

Consistent with Bronfenbrenner’s (1995, 2005) PPCT model, the reviewed evidence supports the centrality of proximal processes in determining refugee child mental health and psychosocial developmental outcomes. Proximal processes are enduring, reciprocal and evolving interactions between the developing child and people, especially ‘significant others’, symbols and objects in their immediate environment (Bronfenbrenner, 1995, 2005). While peers and others in the child's immediate environment can be of relevance in this regard, this highlights the pertinence of focusing on parent–child interaction and parenting behaviours within the reconfigured family life of refugee children (Beiser et al., 2015; Fazel & Betancourt, 2018).

Quality of parent–child relationships (proximal processes) can both enhance and inhibit a protective environment for children (Bermudez et al., 2018). A number of studies point to the effect of warmth, supports, responsivity and stability of parent–child interaction in determining positive mental health outcomes in refugee children (Bermudez et al., 2018; Bettmann & Olson‐Morrison, 2018; Eruyar et al., 2020; McGregor et al., 2016; Nasıroğlu et al., 2018; Oppedal & Idsoe, 2015; Zwi et al., 2018). Parents can increase children’s sense of security and safety by providing emotional warmth and supports, material resources and advice on how to avoid risky situations (Bermudez et al., 2018). Responsive and stable parent–child interactions (proximal processes) are linked with post‐traumatic growth and mitigating the impact of atrocities on children (Sleijpen at al., 2016).

Evidence also indicates that persistent exposure to stressors from multi‐layered developmental contexts exerts a negative effect on children’s emotional well‐being and psychosocial adjustment via parental psychopathology (familial factors), inter‐parental conflict (familial factor) and low quality of parent–child interactions (proximal processes) (Bryant et al., 2018; Dalgård & Montgomery, 2017; Eruyar, Maltby, & Vostanis, 2018, 2020; Panter‐Brick et al., 2015; Van Ee et al., 2013; Stark et al., 2015). Prolonged detention, poor sanitation, adverse economic conditions, hunger, trauma and conflict in the larger system of refugee settlements negatively influence a family system functionality, with members of the family experiencing norms of violence and inter‐parental conflict (Afifi et al., 2019; Dalgård & Montgomery, 2017; McGregor et al., 2016; Sim, Fazel, et al., 2018; Stark et a., 2015).

According to emotional security theory, high levels of parental stress and inter‐parental conflict threaten children’s sense of security and psychosocial adjustment (Cummings & Davies, 2010). Refugee families high in interpersonal conflict may become overloaded with stress. Stress fosters more conflict and conflict elevates stress levels in parents. Accumulated tension from parental mental health and interpersonal conflict often spills over into parent–child interaction and manifests itself in the form of harsh or punitive parenting behaviours (proximal processes); ultimately, negatively impacting on child psychosocial developmental outcomes (Dalgård & Montgomery, 2017; Eruyar, Maltby, & Vostanis, 2018, 2020; Lauritzen & Sivertsen, 2012; Van Ee et al., 2013; Panter‐Brick et al., 2015). Therefore, addressing mental health and psychosocial needs of refugee children requires thorough consideration of the mental health cascade across generations and the cumulative adversities that can negatively impact on proximal processes within a family system. These findings call for family‐centred interventions targeting parental stress levels, promoting parental coping and positive parenting practices. Improvements in family cohesive relationships may support achieving optimal psychosocial and mental health outcomes in refugee children. Similar principles must be applied in the design of supportive services for unaccompanied minors.

### Child agency: demand, resource, force characteristics

Bronfenbrenner (1995, 2005) proposes that individual factors – demand (gender, race, ethnicity), resource (the mental and emotional resources formed through past experiences, skills, intelligence) and force characteristics (temperament, motivation, and persistence) represent child agency in interacting with time, contextual factors and proximal processes and shaping developmental outcomes. Consistent with this view, we identified consistent evidence for contextual and proximal processes within the ecology of refugee children vary as a function of child agency.

Each refugee child is characterised with unique agency (Smith et al., 2015). Events, memories, emotions and experiences can either buffer or worsen the effects of adverse experiences as a function of children’s emotionality (resource), coping and self‐regulation strategies (force characteristics), adaptability (force characteristics), distractibility and attention span (force characteristics) (Akiyama et al., 2013; Buchegger‐Traxler & Sirsch, 2012; Çeri & Nasiroğlu, 2018; Nasıroğlu et al., 2018; Oppedal et al., 2018; Panter‐Brick et al., 2015; Vervliet et al., 2014; Veronese, Pepe, Jaradah, Al Muranak, et al., 2017; Veronese, Pepe, Jaradah, Murannak, et al., 2017). For instance, reports suggest differences in prevalence of psychopathology in refuge children by gender (demand factor), whereas girls appear more negatively affected by adverse experiences than boys (Akiyama et al., 2013; Buchegger‐Traxler & Sirsch, 2012; Çeri & Nasiroğlu, 2018; Nasıroğlu et al., 2018; Oppedal et al., 2018; Panter‐Brick et al., 2015; Vervliet et al., 2014). It is also established that girls appear to prefer intimate and domestic forms of agency (Boxer et al., 2013) and are more prone to exhibit internalization problems in the face of adversity (Kolltveit et al., 2012). Internalization problems may manifest in girls as emotional dysfunction (Kolltveit et al., 2012), dissatisfaction with quality of life, feelings of hopelessness (Nasıroğlu et al., 2018), adjustment difficulties and lower neighbourhood attachment (Buchegger‐Traxler & Sirsch, 2012). Boys, on the other hand, tend to portray agency as living in and populating external spaces (Boxer et al., 2013) and exhibit more conduct and hyperactivity problems when faced with adversity (Kolltveit et al., 2012). Boys therefore may present with vague or somatic symptoms – poor appetite, sleep difficulties, oppositional or aggressive behaviour (Mace et al., 2014) which may mask the actual magnitude of psychopathology.

Nonetheless, in early resettlement many children are able to experience positive adaptational and existential outcomes following adverse experiences (Aitcheson et al., 2017; Mace et al., 2014; McGregor et al., 2016; Veronese et al., 2012). Children may become insightful, self‐reflective on their emotional needs and functioning, experience heightened appreciation for life, spiritual and personal growth (Meyerson, Grant, Carter, & Kilmer, 2011).

Although children bring resource and force characteristics with them and may present as ‘resilient’ (Aitcheson et al., 2017; Mace et al., 2014; McGregor et al., 2016; Veronese et al., 2012), the way social and physical aspects of their environment respond (proximal processes) are an important influence on their psychosocial trajectories. For instance, in females residing in camps the adjustment difficulties, lower neighbourhood attachment, engagement in risky behaviour (e.g. transactional sex) is partly influenced by social and physical distancing caused by peer and adult discrimination (proximal processes), poverty and poor living conditions (contextual factors) (Bermudez et al., 2018; Buchegger‐Traxler & Sirsch, 2012; Sim, Fazel, et al., 2018). On the other hand, unaccompanied refugee minors who get adequate support from peers and detention centre staff (proximal process) report lower levels of anxiety, worries and depression while waiting for lengthy asylum process (institutional factors) at resettlement (Eriksson & Rundgren, 2019; Groark et al., 2011; Thommessen et al., 2015). Refugee children therefore make better use of their adaptational abilities and may maintain a strong sense of self when adequately supported by ‘significant others’ (e.g. parents, peers, adults, support staff), symbols (e.g. belief system, language, culture), and objects (e.g. supportive policies and institutions) in their daily environment.

### Implications for practice

The preceding analysis has identified empirical evidence of a wide range of factors having influence on the mental health and well‐being of refugee children. Interventions – whether focused on policy or programming – that moderate risk factors or promote protective factors clearly stand to secure positive impact. However, grounding the preceding analysis within the PPCT frame suggests three wider principles shaping intervention planning.

First, the systems framing points to the potential for interventions at one systemic level to influence factors at other levels and, further, the potential value of identifying and synergising such linkages. Structural interventions in camp settings, for example in relation to shelter, are likely to have direct impacts on children’s well‐being. However, the benefits may also support community‐, school‐ and household‐based factors supporting children in this environment, especially if such benefits are actively anticipated and cultivated (Ager, Annan & Panter‐Brick, 2013). An increasing number of studies are now adopting an ecosystems understanding of intervention of this form, this anticipation of systems linkages distinguishing it from a more siloed approach. The Welcoming Cities movement in the USA (Welcoming America, 2017) provides a vivid example of how policy intervention at the exosystemic level can facilitate change in multiple parts of ecosystem (including housing, healthcare, schooling, community relations and employment) into which a refugee child is resettling (McDaniel, Rodriguez, & Wang, 2019).

Second, while a diverse range of influences on child refugee mental health may be identified, PPCT analysis points to the crucial role of proximal processes, and thus the central relevance of interventions addressing parenting. The general upsurge in focus on parenting interventions of the last decade (Hodes and Vostanis, 2019) is to be welcomed. However, specific supportive interventions are needed which address proximal processes within refugee households under acute migratory or resettlement stressors. Psychoeducational interventions shaped by an understanding of processes of acculturation and the potential impacts of past traumatic exposure (e.g. Bjorn, Boden, Sydsjo, & Gustaffson, 2013; Fazel & Betancourt, 2018; Osman, Flacking, Schon & Klingberg‐Allvin, 2017; Slobodin & de Jong, 2015) are likely to be particularly effective.

Third, Bronfenbrenner’s formulation of the PPCT model was substantially driven by a concern to ensure children’s development is not understood as solely socially determined but also reflecting the agency of the child. As noted above, our analysis suggests that there is an important place for child agency in intervention design for refugee children. We see significant value here in Ungar’s formulation of resilience as ‘the capacity of a biopsychosocial system (this can include an individual person, a family or a community) to navigate to the resources necessary to sustain positive functioning under stress, as well as the capacity of systems to negotiate for resources to be provided in ways that are experienced as meaningful’ (Ungar, 2011). This understanding emphasises the value of interventions which strengthen capacity for navigation towards, and securing of, personal, familial and community resources. Strengthening such capacity – in line with developmental age – should be the central focus of preventive interventions regarding child refugee mental health and well‐being. Noting the interdependencies discussed above, this will often be best accomplished in the context of multi‐layered interventions addressing both contextual and individual factors influencing adjustment.

### Limitations

Several limitations should be noted. Refugee children are a widely diverse group. Elaborations presented in this review do not distinguish between specific needs of high‐risk refugee populations, for example, unaccompanied refugee minors, trafficked and undocumented children, former child soldiers and those with a history of torture, who might need more urgent and tailored mental health support interventions. We collate evidence from studies in widely differing social, economic and cultural contexts, including children in protracted camp settlements and those resettled in high‐income host countries, within the same analytic frame. This allows us to observe trends in common across these diverse experiences, but should not be taken to underplay the significance of differences in these contexts. Evidence is collated from peer‐reviewed articles indexed in four electronic databases in English. This may have introduced publication and language biases. Although these biases have received much less consideration in narrative reviews than systematic reviews and meta‐analyses, their potential effects should be acknowledged. Most importantly, there are constraints associated with the use of a framework of risk and protective factors to organise literature search and data extraction. Although we adopted clear definitions for these terms, we acknowledge that selected studies drew on a wide range of alternative theoretical framings for these constructs. Further, the relative impact on developmental trajectories of the risk and protective factors considered should not be implied: this has not been formally assessed and – as indicated by the analysis – will, indeed, vary widely across contexts and time‐course.

## Conclusion

The study notes the burgeoning literature on mental health and psychosocial well‐being of refugee children, significantly deepening our understanding of factors shaping experience and outcomes. A bioecological framing of the varied risks and protective factors influencing refugee children’s well‐being serves as a suitable frame for collating this evidence, especially with regard to processes of their interaction. Bronfenbrenner’s later PPCT model serves to highlight the interaction of historical time and developmental age, the pivotal role of proximal processes and the influence of child agency in determining outcomes. Informed by this framing of the evidence base, mental health‐promoting interventions should place greater emphasis on promoting positive proximal processes operating between an active, evolving refugee child and ‘significant others’ (parents, peers, adults, teachers and support staff), symbols (belief system, language, culture) and objects (policies and institutions) in their daily environment. These interventions targeting proximal processes are necessary if appropriate actions to address risks and stressors at individual, community, school, institution and policy levels are to have traction on mental health and psychosocial well‐being.
